# Carnosine Impedes PDGF-Stimulated Proliferation and Migration of Vascular Smooth Muscle Cells In Vitro and Sprout Outgrowth Ex Vivo

**DOI:** 10.3390/nu12092697

**Published:** 2020-09-03

**Authors:** Byungdoo Hwang, Jun-Hui Song, Sung Lyea Park, Jee Taek Kim, Wun-Jae Kim, Sung-Kwon Moon

**Affiliations:** 1 Department of Food and Nutrition, Chung-Ang University, 4726 Seodong-Daero, Daedeok-Myeon, Anseong 17546, Korea; byungdoo0409@naver.com (B.H.); goodabc123@cau.ac.kr (J.-H.S.); ajzzang9090@hanmail.net (S.L.P.); 2 Department of Ophthalmology, College of Medicine, Chung-Ang University, Heukseok-ro, Dongjak-gu, Seoul 84, Korea; jeetaek@cau.ac.kr; 3 Department of Urology, Chungbuk National University, Cheongju 361-763, Korea; wjkim@chungbuk.ac.kr

**Keywords:** carnosine, vascular smooth muscle cells, platelet-derived growth factor, proliferation, migration, aortic ring ex vivo

## Abstract

Carnosine, a naturally producing dipeptide, exhibits various beneficial effects. However, the possible role of carnosine in vascular disorders associated with pathological conditions, including proliferation and migration of vascular smooth muscle cells (VSMCs), largely remains unrevealed. Here, we investigated the regulatory role and mechanism of carnosine in platelet-derived growth factor (PDGF)-induced VSMCs. Carnosine inhibited the proliferation of PDGF-induced VSMCs without any cytotoxic effects. Carnosine treatment also induced G1-phase cell cycle arrest by causing a p21WAF1-mediated reduction in the expression of both cyclin-dependent kinases (CDKs) and cyclins in PDGF-treated VSMCs. Carnosine treatment suppressed c-Jun N-terminal kinase (JNK) phosphorylation in PDGF-stimulated signaling. Additionally, carnosine significantly prevented the migration of VSMCs exposed to PDGF. Carnosine abolished matrix metalloproteinase (MMP)-9 activity via reduced transcriptional binding activity of NF-κB, Sp-1, and AP-1 motifs in PDGF-treated VSMCs. Moreover, using aortic assay ex vivo, it was observed that carnosine addition attenuated PDGF-stimulated sprout outgrowth of VSMCs. Taken together, these results demonstrated that carnosine impeded the proliferation and migration of PDGF-stimulated VSMCs by regulating cell cycle machinery, JNK signaling, and transcription factor-mediated MMP-9 activity as well as prevented ex vivo sprout outgrowth of blood vessels. Thus, carnosine may be a potential candidate for preventing vascular proliferative disease.

## 1. Introduction

Cardiovascular disease (CVD) is one of the leading causes of death worldwide [1]. Uncontrolled vascular smooth muscle cell (VSMC) proliferation and migration is a critical event involved in the development of intimal hyperplasia, which produced a vascular lesion that resulted in vascular disorders including re-stenosis and atherosclerosis [2,3]. Among numerous pro-inflammatory cytokines and growth factors, platelet-derived growth factor (PDGF) strongly stimulated the proliferative and migratory potential of VSMCs [4], resulting in an increased VSMC proliferation and migration from the media to intima [5]. Therefore, identifying candidates that can repress the PDGF-mediated proliferation and migration of VSMCs has been considered to be a good strategy to prevent or treat proliferative vascular disease [2].

PDGF-stimulated VSMCs response may involve early intracellular signaling pathways and critical regulatory molecules such as cell cycle-associated proteins, mitogen-activated protein kinases (MAPKs) and protein kinase b (AKT) signaling molecules, matrix metalloproteinase (MMP) proteins, and transcription factor-mediated migration [6,7,8,9]. During vascular remodeling, PDGF induces VSMC proliferation via multiple signaling cascade pathways including the MAPKs (JNK, p38MAPK, and ERK1/2) and AKT phosphorylation [6,7,8]. After the vascular injury, VSMCs transmit from G1- to S-cell cycle in response to treatment with PDGF [8,9]. Progression of the cell cycle is directly modulated via the activation of cyclin/CDK complexes [8,9]. The kinase activity of cyclin/CDK complexes is also coordinated by negative cell cycle inhibitors, such as p21WAF1 and p27KIP1 [9,10].

Extracellular matrix (ECM) remodeling requires the expression of MMPs that degrade basement membranes and facilitate neointima formation [10]. ECM breakdown by MMPs induces the migration of VSMCs followed by that of several stimulants [8,10,11,12,13]. MMPs are zinc-dependent endopeptidases that regulate various biological properties linked with vascular matrix remodeling [10]. In particular, MMP-9 is essential for the progression and development of proliferative vascular diseases [8,11,12,13]. In an animal model, MMP-9-deficient mice exhibited a reduced vessel sprouting and neointimal thickening owing to decreased migration and proliferation of VSMCs [11,12]. Additionally, expression of MMP-9 is significantly upregulated by the addition of growth factors including PDGF [8]. The promoter of MMP-9 gene contains functional multiple binding sites including three AP-1 sites, an Sp-1 site, an Ets site, a retinoblastoma element, and a nonconsensus NF-κB [13,14,15,16]. It has also been recognized that MMP-9 expression is controlled by transcriptional binding activities of AP-1, NF-κB, and Sp-1 motifs [13,14,15,16].

Carnosine (β-alanyl-l-histidine) is a natural dipeptide that is spontaneously synthesized by our body [17] and is distributed in nerve tissues (0.7–2.0 mM) and skeletal muscles (20 mM) at high concentrations [18]. The potential of carnosine as a pharmacological agent, including its anti-cancer, anti-ocular, anti-neural, anti-ischemic, and anti-diabetic effects, has been widely suggested in various model systems [18]. A recent study found that carnosine also inhibited lipid oxidation and lipid peroxidation product generation in an ApoE-null mice model [19]. However, the exact molecular mechanism of carnosine-mediated anti-vascular diseases remains unclear. Thus, to our knowledge, the regulatory molecular mechanism of carnosine in the PDGF-stimulated VSMC proliferation and migration in vitro and ex vivo has been investigated for the first time in our study.

## 2. Materials and Methods

### 2.1. Materials

l-carnosine was obtained from Sigma-Aldrich (St. Louis, MI, USA). Polyclonal antibodies of ERK, JNK, AKT, p38MAPK, phospho-ERK, phospho-JNK, phospho-p38MAPK, phospho-AKT, MMP-2, and MMP-9 were purchased from Cell Signaling Technology (Danvers, MA, USA). Polyclonal antibodies against CDK2, CDK4, cyclin E, cyclin D1, p21WAF1, p27KIP1, and glyceraldehyde 3-phosphate dehydrogenase (GAPDH), and p53 were obtained from Santa Cruz Biotechnology (Santa Cruz, CA, USA). SP600125 (JNK inhibitor), MMP-2 inhibitor, and MMP-9 inhibitor were also purchased from Santa Cruz Biotechnology (Santa Cruz, CA, USA).

### 2.2. Cell Culture

The aortas of Sprague–Dawley rats were used to obtain isolation of VSMCs as demonstrated previously [8], and maintained in 1× DMEM containing 10% fetal bovine serum (FBS), 2 mM glutamine, 50 mg/mL amphotericin-B, and 50 mg/mL gentamycin at 37 °C in a 5% humidified CO_2_ incubator. The cells were used between passages 5 and 8. For use in all experiments, the cells were grown until they attained 70–80% confluence and made synchronization via serum-starvation for at least 24 h in FBS-free DMEM.

### 2.3. Cell Viability Assay and Cell Counting Assay

Cell viability was determined using both the 3-(4,5-dimethylthiazol-2-yl)-2,5-diphenyltetrazolium bromide (MTT) and viable cell counting assays. For the MTT assay, 6 × 10^3^ cells were cultured per well in 96-well plates, followed by a 45-min preincubation with carnosine at concentrations of 10, 15, and 20 mM and subsequently treated with PDGF (20 ng/mL). After a 24-h incubation period, the medium was changed with fresh medium supplemented with 10 μL MTT (5 mg/mL). After allowing the reaction to occur for 1 h, the medium was removed and 100 μL dimethyl sulfoxide (DMSO) was then added to each well. Cell viability was assessed via detection of the absorbance at 540 nm using a fluorescence microplate reader. For viable cell counting, detached cells (50 μL) were combined with 50 μL of 0.4% trypan blue (Sigma-Aldrich). After gentle pipetting, the blue-stained dead cells were counted using a hemocytometer. Viable cells were measured by the following equation: viable cells (%) = [1.00 − (number of stained cells ÷ number of total cells)] × 100.

### 2.4. Cell Cycle Analysis

Serum-starved VSMCs were pre-incubated with carnosine at concentrations of 10, 15, and 20 mM for 45 min, and then exposed to PDGF (20 ng/mL) for 24 h. The cells were then trypsinized, collected, and washed twice with phosphate-buffered saline (PBS). After centrifugation at 1000× *g*, the cells were fixed using 5 mL of ice-cold ethanol (70% (*v*/*v*)) at 4 °C overnight. After centrifugation, cell pellets were incubated with 1 mL PBS incorporating propidium iodide (PI, 100 μg/mL) and RNase A (100 μg/mL) at 37 °C for 30 min. Cell cycle analysis was subjected to analysis using a flow cytometer (FACStar™, Becton Dickinson, Franklin Lakes, NJ, USA) equipped with FlowJo version 10 software (Tree Star, Ashland, OR, USA).

### 2.5. Immunoblot

The cells were lysed in 250 µL of lysis buffer (containing, in mM/L, 50 HEPES (pH 7.5), 150 mM NaCl, 1 mM EDTA, 2.5 mM EGTA, 1 mM DTT, 10 mM β-glycerophosphate, 1 mM NaF, 0.1 mM Na_3_VO_4_, and 0.1 mM phenylmethylsulfonylfluoride, 0.1% Tween-20, 10% glycerol, 2 µg/mL aprotinin, and 10 μg/mL leupeptin) followed by incubation for 30 min at 4 °C. After centrifugation of lysates at 12,000 rpm for 20 min at 4 °C, the cell supernatant was quantified via Bradford assay. Equal amounts (15 μg) of the total protein samples were separated by 10% sodium dodecyl sulfate-polyacrylamide gel electrophoresis (SDS-PAGE) and transferred to nitrocellulose membranes (Hybond, GE Healthcare Bio-Sciences, Marlborough, MA, USA). The membranes were incubated overnight with specific primary antibodies at 4 °C, followed by 1-h treatment with horseradish peroxidase-conjugated secondary antibodies to enable band detection. Immunoreactive protein expressions were visualized using a chemiluminescence detection system (Hybond, GE Healthcare Bio-Sciences, Marlborough, MA, USA). Similar experiments were conducted at least three independent times.

### 2.6. Immunoprecipitation

The cell lysates were reacted overnight with indicated antibodies at a concentration of 4 μg/mL at 4 °C. Immunocomplexes were precipitated by incubating protein A-Sepharose beads for 2 h at 4 °C. Immunoprecipitates were washed with lysis buffer twice, followed by resuspension in SDS-PAGE sample buffer including β-mercaptoethanol. The protein samples were estimated via immunoblotting.

### 2.7. JNK Inhibition (SP600125) Experiment

Serum-starved cells were pre-treated with SP600125 for 40 min, then followed by treatment with indicated concentration of PDGF for 10 min. Thereafter, cells were collected, and the ratio of the phosphorylated form to the non-phosphorylated protein of JNK was determined by immunoblot. For MTT assay, cell counting assay, cell cycle analysis, wound-healing assay, and transwell migration assay, cells were pre-incubated with SP600125 for 40 min, prior to stimulation of PDGF for 24 h.

### 2.8. Wound-Healing Assay

Serum-starved cells were cultured to attain a 90% confluence in six-well plates and subsequently damaged using the tip of a pipette. For prevention of cell proliferation, the cells were reacted with mitomycin C (10 μg/mL) for 1 h, followed by incubation with indicated concentrations of carnosine (10, 15, and 20 mM) in the absence or presence of PDGF (20 ng/mL). After a 24-h incubation, the migrated area was captured and measured using a phase-contrast microscope.

### 2.9. Transwell Migration Assay

A transwell migration assay was conducted using the 6-well transwell plates with a transwell Boyden Chamber membrane of 8.0-μm pore size (Corning Inc., Corning, NY, USA). The serum-starved cells were incubated in serum-free DMEM + mitomycin C (10 μg/mL) supplemented with carnosine either alone or together with PDGF, and then they were seeded into each of the upper sections of the chamber plates for 24 h, allowing cellular invasiveness through the membrane. The cells from the upper side of the membrane were scraped off with cotton swabs, and invaded cells were fixed in 90% EtOH and stained with crystal violet (0.1%). The relative invasive cells were counted by the number of invaded cells under a microscope across six randomly selected areas at 200× magnification per each well.

### 2.10. Zymography

Conditioned medium was prepared and separated by 8% SDS-PAGE containing gelatin (1 mg/mL). The gels were washed twice using a 2.5% Triton X-100 to remove SDS for 2 h at room temperature, followed by reaction with an incubation buffer (150 mM NaCl, 10 mM CaCl_2_, and 50 mM Tris–HCl, pH 7.5) at 37 °C overnight. The gels were stained with 0.2% Coomassie brilliant blue, and subsequently de-stained with a buffer containing 50% methanol and 5% acetic acid. Gelatinase activity was visible as horizontal white bands against a dark blue background.

### 2.11. Nuclear Extracts and Electrophoretic Mobility Shift Assay (EMSA)

EMSA experiments were conducted using nuclear extracts as described earlier [8,13]. Cells were obtained, washed, and then mixed in a buffer (10 mM KCl, 10 mM Hepes (pH 7.9), 0.1 mM EGTA, 0.1 mM EDTA, 0.5 mM PMSF, and 1 mM DTT) for 15 min at 4 °C. The cells were vortexed with 0.5% Nonidet NP-40, and then a nuclear pellet was collected via centrifugation; this was followed by extraction in a buffer (20 mM Hepes pH 7.9, 0.4 M NaCl, 1 mM EGTA, 1 mM EDTA, 1 mM PMSF, and 1 mM DTT) for 15 min at 4 °C. The oligonucleotide sequences were listed as follows: Sp-1, GCCCATTCCTTCCGCCCCCAGATGAAGCAG; NF-κB, CAGTGGAATTCCCCAGCC; and AP-1, CTGACCCCTGAGTCAGCACTT. The binding mixture for reaction was incubated at 4 °C for 20 min in a buffer (25 mM HEPES buffer pH 7.9, 0.5 mM EDTA, 0.5 mM DTT, 0.05 M NaCl, and 2.5% glycerol) containing 2 µg of poly dI/dC and 5 fmol (2 × 10^4^ cpm) of a oligonucleotide containing Klenow end-labeled with [γ-^32^P] ATP, which spanned the DNA-binding site corresponding to the promoter of MMP-9. For competition lane, the aliquots of nuclear extract (10–20 µg) were pre-incubated by using a 100-fold excess of an unlabeled oligonucleotide 4 °C for 30 min before addition of labeled oligonucleotide. The reaction solution was electrophoretically resolved at 4 °C in a 6% polyacrylamide gel by a TBE running buffer (89 mM Tris, 89 mM boric acid, and 1 mM EDTA). The gel was then rinsed, dried, and followed by exposure of an X-ray film overnight. The bands were visualized and estimated using ImagePro Plus 6.0 software (Media Cybernetics, Bethesda, MD, USA).

### 2.12. Aortic Ring Assay

The proliferation and migration of VSMC ex vivo were analyzed by an aortic ring assay via Matrigel, as previously described [20]. Briefly, the adventitium and endothelium of the thoracic aorta from four male Sprague–Dawley rats (5-week-old) were removed carefully via a fine surgical method, and 1-mm thick rings were cut and sectioned. The aortic rings were rinsed and embedded onto Matrigel-coated 24-well plates, followed by pre-incubation with FBS-free DMEM supplemented with carnosine for 45 min and subsequent treatment with PDGF (20 ng/mL). After a 5-day culture, the outgrowth of sprout formation of VSMCs from aortic rings was photographed via a ZEISS microscope (Carl Zeiss, Oberkochen, Germany), and the length of the sprouts was evaluated by the use of ImagePro Plus 6.0 software (Media Cybernetics, Bethesda, MD, USA). All animal experiments were reviewed and approved by the institutional Animal Care and Use Committee of Chungbuk National University.

### 2.13. Statistical Analysis

Data were represented as mean ± SE values. The significant differences between groups were analyzed using factorial ANOVA analysis and Fisher’s least significant difference test. Statistical differences were considered significant at a *p* value of <0.05.

## 3. Results

### 3.1. Carnosine Inhibited PDGF-Induced Proliferation of VSMCs

**Figure 1:**
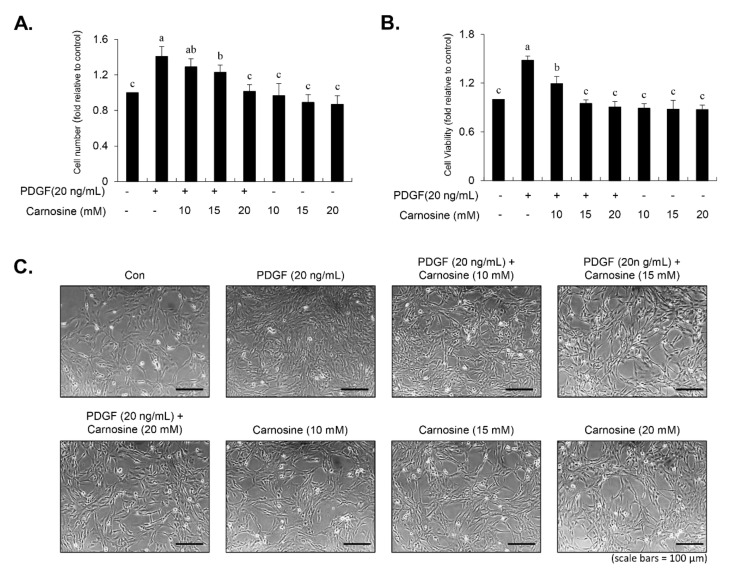
Carnosine inhibited the proliferation of platelet-derived growth factor (PDGF)-induced vascular smooth muscle cells (VSMCs). After 24-h serum starvation, VSMCs were treated with indicated carnosine concentrations for 45 min, and then stimulated with or without PDGF (20 ng/mL) for 24 h. (**A**) Cell proliferation was analyzed by cell counting. (**B**) Based on the 3-(4,5-dimethylthiazol-2-yl)-2,5-diphenyltetrazolium bromide (MTT) assay, cell viability was measured. (**C**) Morphologic changes in PDGF-treated VSMCs when treated with the indicated concentrations of carnosine were photographed. All data are expressed as the mean ± SE of three experiments. The different letters indicate significant differences between the groups (*p* < 0.05).

Using the trypan blue exclusion method, we examined the effect of carnosine in PDGF-induced VSMC proliferation. VSMCs were pre-incubated with various concentrations of carnosine for 45 min, then followed by PDGF treatment for 24 h. As shown in Figure 1A, carnosine treatment suppressed the PDGF-stimulated proliferation of VSMCs in a concentration-dependent manner. Proliferative potential of PDGF-induced VSMCs was reversed to a control level without cellular toxicity in the presence of carnosine at a concentration of 20 mM (Figure 1A). Treatment with vehicle did not affect cell death (Figure 1A). Cell proliferation markedly decreased at carnosine concentrations of 20 mM (Figure 1A). Similar patterns of results were observed in the MTT assay (Figure 1B). Carnosine treatment reduced the number of PDGF-treated VSMCs, as observed under a microscope (Figure 1C). These results indicated that carnosine repressed the proliferation of PDGF-stimulated VSMCs without causing any cellular toxicity.

### 3.2. G1-Phase Arrest of the Cell Cycle by Suppressing the Level of CDKs/Cyclins Expression in PDGF-Treated VSMCs

**Figure 2:**
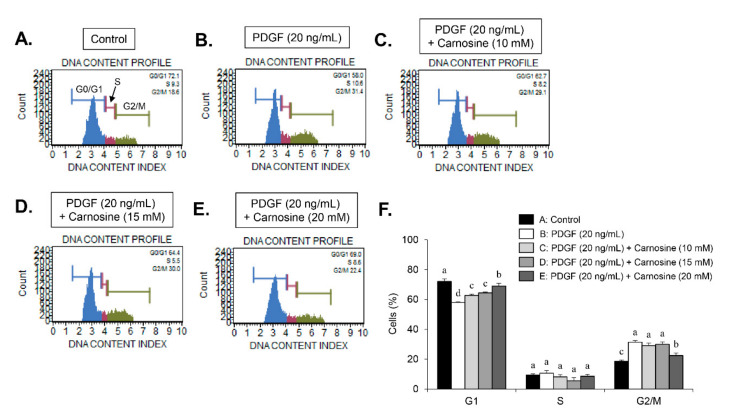
Carnosine-induced G1-phase cell cycle arrest in PDGF-treated VSMCs. VSMCs were pre-cultured with various concentrations of carnosine for 45 min and then incubated with PDGF (20 ng/mL) for 24 h. FACS histograms are shown for (**A**) control (Con), (**B**) PDGF alone, (**C**) 10 mM carnosine with PDGF, (**D**) 15 mM carnosine with PDGF, (**E**) 20 mM carnosine with PDGF, and (**F**) cell cycle distribution is expressed as the percentages of cells at the G1-, S-, and G2/M-phases. Values are expressed as mean ± SD values of three independent experiments. The different letters indicate significant differences between the groups (*p* < 0.05).

**Figure 3:**
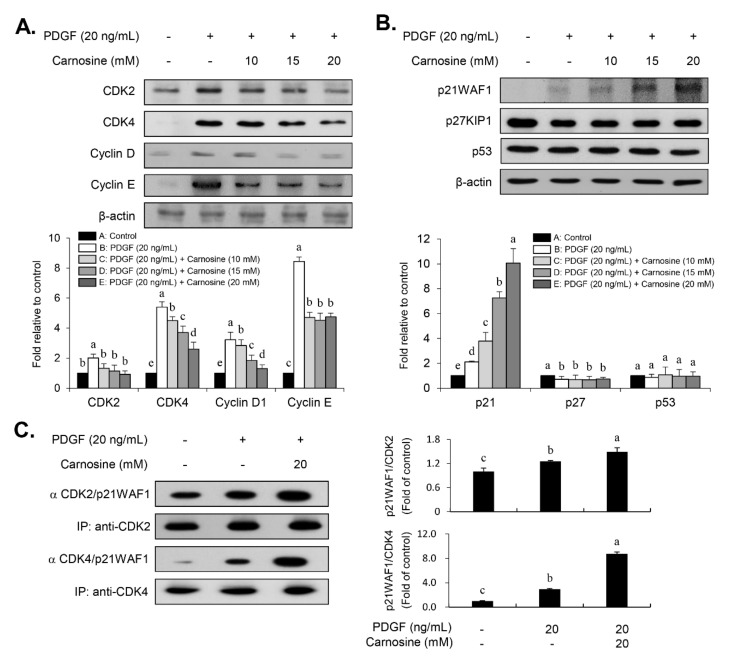
Carnosine repressed the expression levels of CDKs and cyclins and increased the expression level of p21WAF1 in PDGF-treated VSMCs. The cell lysates were analyzed by immunoblots with antibodies specific for (**A**) CDK2, CDK4, cyclin D1, and cyclin E as well as (**B**) p21WAF1, p27KIP1 and p53. Actin was used as an internal control. (**C**) Cell lysates were immunoprecipitated with anti-CDK2 and anti-CDK4 antibodies, and then immunoblotted with anti-p21WAF1 antibody. All graphs exhibit the relative expression of the corresponding proteins on the blots. Values are expressed as mean ± SD values of three independent experiments. The different letters indicate significant differences between the groups (*p* < 0.05).

We examined whether carnosine would induce the PDGF-stimulated inhibition of VSMC proliferation by arresting a specific cell cycle phase using flow cytometry. PDGF treatment increased the cell number of S-phase and G2/M-phase as well as simultaneously decreased the cell number in G1-phase population (Figure 2A,B,F). Treatment with carnosine increased the population of G1-phase in PDGF-treated cells and reduced the fraction of S- and G2/M-phase in PDGF-treated cells (Figure 2A–F). These results indicate that carnosine can induce G1-phase cell cycle arrest and impede the S- and G2/M-phase entry in PDGF-stimulated VSMCs, which results in the reduction of cell proliferation. Subsequently, we investigated the expression levels of G1-phase cell cycle-related proteins to determine the cell cycle regulatory mechanism of carnosine in PDGF-induced VSMCs. Thus, the expression levels of positive G1-phase regulators, such as CDK2, CDK4, cyclin D1, and cyclin E, were determined in VSMCs. PDGF treatment markedly upregulated the expression levels of CDK2, CDK4, cyclin D1, and cyclin E (Figure 3A). Increased expression levels of CDK2, CDK4, cyclin D1, and cyclin E by PDGF were attenuated in carnosine-treated VSMCs (Figure 3A). These results suggested that carnosine inhibited PDGF-promoted cell cycle progression in the S and G2/M phases via induction of cell cycle arrest at G1-phase by downregulating CDKs/cyclins in VSMCs.

### 3.3. Carnosine Stimulated G1-Phase Cell Cycle Arrest via CDKs Binding with p21WAF1 in PDGF-Treated VSMCs

To further investigate the exact mechanism of G1-phase cell cycle arrest by carnosine in PDGF-treated VSMCs, the cellular levels of negative cell cycle modulators, including CDK inhibitors (CDKIs, p21WAF1, and p27KIP1) and tumor suppressors (p53), were employed. PDGF promoted the p21WAF1 expression and reduced the p27KIP1 expression in VSMCs (Figure 3B). The expression level of p53 was not changed by PDGF (Figure 3B). Interestingly, carnosine treatment enhanced p21WAF1 expression induced by PDGF in VSMCs (Figure 3B). However, the reduction in p27KIP1 expression induced by PDGF remained unaffected by the addition of carnosine (Figure 3B). Carnosine treatment had no effect on the level of p53 expression in PDGF-treated VSMCs (Figure 3B). Subsequently, we examined whether carnosine mediated the inhibition of CDKs owing to p21WAF1 expression. Cell lysates were used to perform the immunoprecipitation experiment in carnosine-treated VSMCs stimulated by PDGF. PDGF treatment augmented the interaction level of p21WAF1 bound to CDK4 and CDK2 in VSMCs (Figure 3C). Additionally, carnosine treatment further enhanced the amount of p21WAF1 bound to CDK4 and CDK2 in PDGF-stimulated VSMCs (Figure 3C). These results indicated that carnosine induced G1-phase cell cycle arrest via induction of CDKs binding to p21WAF1 in PDGF-treated VSMCs and thereby caused the downregulation of CDKs expression.

### 3.4. Carnosine Inhibited the Phosphorylation of JNK in PDGF-Treated VSMCs

**Figure 4:**
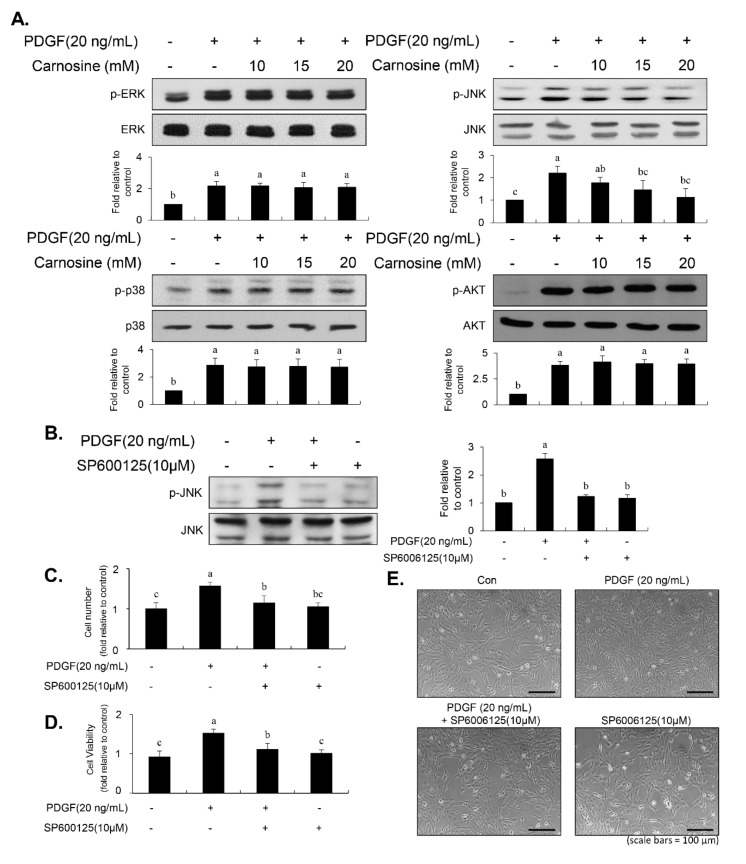
Carnosine suppressed JNK phosphorylation in PDGF-induced VSMCs. (**A**) VSMCs were pre-incubated with carnosine at the concentrations of 10, 15, and 20 mM for 45 min, followed by treatment with PDGF (20 ng/mL) for 10 min. Cell lysates were subjected to immunoblotting with specific antibodies against phospho-ERK1/2, ERK1/2, phospho-p38, p38, phospho-JNK, JNK, AKT, and phospho-AKT. The ratio of the phosphorylated form to the un-phosphorylated protein was estimated and indicated as a fold change compared with that of the control. (**B**–**E**) Cells were pre-treated with SP600125 (10 μM) for 45 min, followed by PDGF treatment for 10 min or 24 h. Immunoblot (**B**), cell counting (**C**), MTT assay (**D**), and morphologic changes (**E**) were evaluated. Each value is expressed as the mean ± SD values of three independent experiments. The different letters indicate significant differences between the groups (*p* < 0.05).

**Figure 5:**
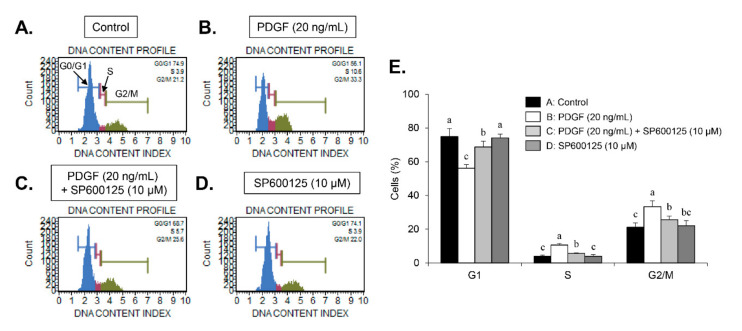
Effect of SP600125 in PDGF-induced reduction of VSMC population at G1-phase of the cell cycle. VSMCs were pre-incubated with SP600125 (10 μM) for 45 min and then treated with PDGF (20 ng/mL) for 24 h. FACS histograms are shown for (**A**) control (Con), (**B**) PDGF alone, (**C**) 10 μM SP600125 with PDGF, (**D**) 10 μM SP600125, and (**E**) cell cycle profile is expressed as the percentages of cell population at the G1-, S-, and G2/M-phases. Values are expressed as mean ± SD values of three independent experiments. The different letters indicate significant differences between the groups (*p* < 0.05).

The early MAPK (ERK1/2, p38MAPK, and JNK) and AKT signaling pathways are deeply associated with the PDGF-induced VSMC proliferation [6,7,8]. We next investigated whether carnosine affect the MAPK and AKT signaling pathways in PDGF-treated VSMCs. PDGF treatment significantly induced the phosphorylation of p38MAPK, ERK1/2, JNK, and AKT in VSMCs (Figure 4A). PDGF-induced phosphorylation of JNK was attenuated by treatment with carnosine (Figure 4A). However, upregulated levels of ERK1/2, p38MAPK, and AKT phosphorylation by PDGF were not inhibited in the presence of carnosine (Figure 4A). We next used SP600125 (JNK inhibitor) to investigate whether inhibition of JNK phosphorylation affects the PDGF-induced proliferation of VSMCs via reduction of population at G1-phase cell cycle. Pretreatment with SP600125 suppressed the JNK phosphorylation induced by PDGF (Figure 4B). As shown in Figure 4C–E, inhibition of JNK signaling reduced proliferation of VSMCs in response to PDGF. In addition, the reduction of cell fraction at G1-phase by PDGF was reversed by pre-treatment with SP600125 (Figure 5A–E). PDGF-induced population of S- and G2/M-phase cell cycle was reduced by SP600125 treatment (Figure 5A–E). These results indicated that carnosine inhibited PDGF-induced VSMC proliferation via up-regulation of G1-phase cell cycle arrest, which was partially due to the suppression of JNK phosphorylation.

### 3.5. Carnosine Suppressed PDGF-Stimulated VSMC Migration

**Figure 6:**
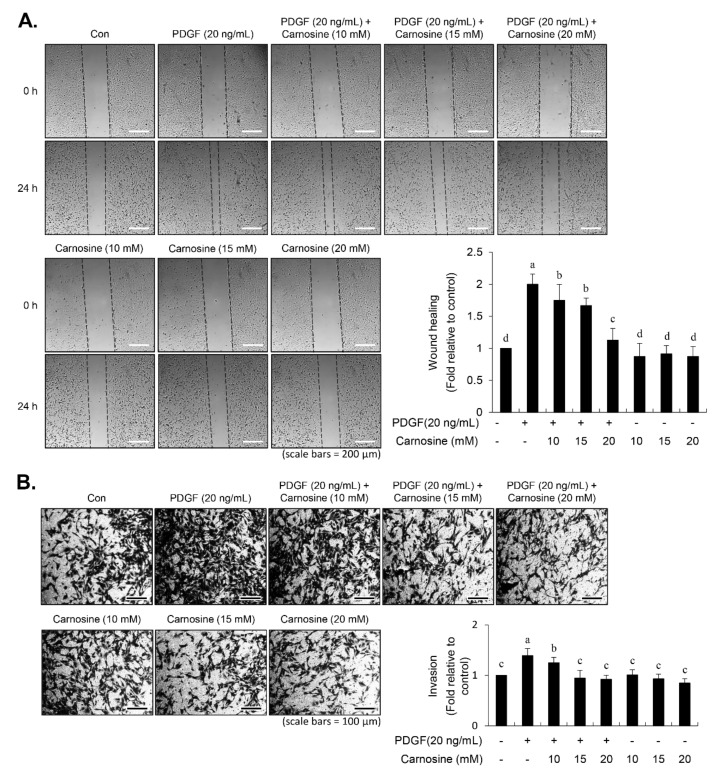
Carnosine abolished the migratory potential of VSMCs induced by PDGF. After 24-h serum starvation, VSMCs were pre-treated with 10, 15, and 20 mM of carnosine for 45 min, and then incubated with or without PDGF (20 ng/mL) for 24 h. (**A**) Wound-healing rate of VSMCs was estimated using phase contrast microscopy. The scratched area in the well is shown as dashed lines. (**B**) VSMCs were seeded in Matrigel-coated transwell plates. Invasive cells were quantified via blind counting under a microscope (40× magnification). The number of transmigrated cells was counted and the averages were measured. The graph shows the fold change relative to the control value. Values are expressed as the mean ± SD values of three independent experiments. The different letters indicate significant differences between the groups (*p* < 0.05).

To investigate the effect of carnosine on the VSMC migration in response to PDGF, wound-healing and transwell migration assays were performed. In the wound-healing assay, a scratch wound was made using the tip of a pipette, followed by addition of PDGF for 24 h. As shown in Figure 6A, PDGF treatment increased VSMC migration. Carnosine treatment caused a significant decrease in PDGF-induced migration of VSMCs (Figure 6A). Moreover, the transwell Boyden Chamber assay confirmed that PDGF treatment of VSMCs increased the number of invaded cells through the transwell membrane chamber (Figure 6B). Carnosine significantly reduced the number of VSMCs that invaded through the transwell plate chamber in the presence of PDGF (Figure 6B). These results suggested that carnosine suppresses the migration of VSMCs that were stimulated by PDGF.

### 3.6. Inhibition of JNK Phosphorylation by SP600125 Repressed the PDGF-Induced Migration

**Figure 7:**
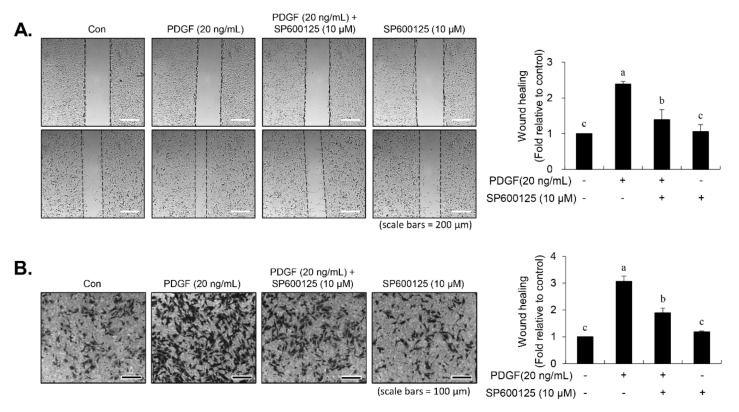
SP600125 inhibited PDGF-stimulated migration of VSMCs. Serum-starved cells were pre-treated for 45 min in the presence of SP600125 (10 μM). Cells were then cultured with PDGF (20 ng/mL) for 24 h. Wound-healing assay (**A**) and transwell migration assay (**B**) were performed as described in Materials and Methods. Values are expressed as the mean ± SD values of three independent experiments. The different letters indicate significant differences between the groups (*p* < 0.05).

To examine the relationship between JNK signaling and migration in PDGF-induced VSMCs, cells were pre-incubated with JNK inhibitor SP600125 for 45 min prior to PDGF stimulation for 24 h. SP600125 treatment inhibited the migratory potential stimulated by PDGF in VSMCs (Figure 7A). In addition, PDGF-induced invasion ability of VSMCs was abolished in the presence of SP600125 (Figure 7B). These data demonstrated that JNK signaling is involved in the PDGF-stimulated migration of VSMCs.

### 3.7. Carnosine Prevented PDGF-Stimulated MMP-9 Expression via Downregulation of NF-κB, Sp-1, and AP-1 Binding Activities

**Figure 8:**
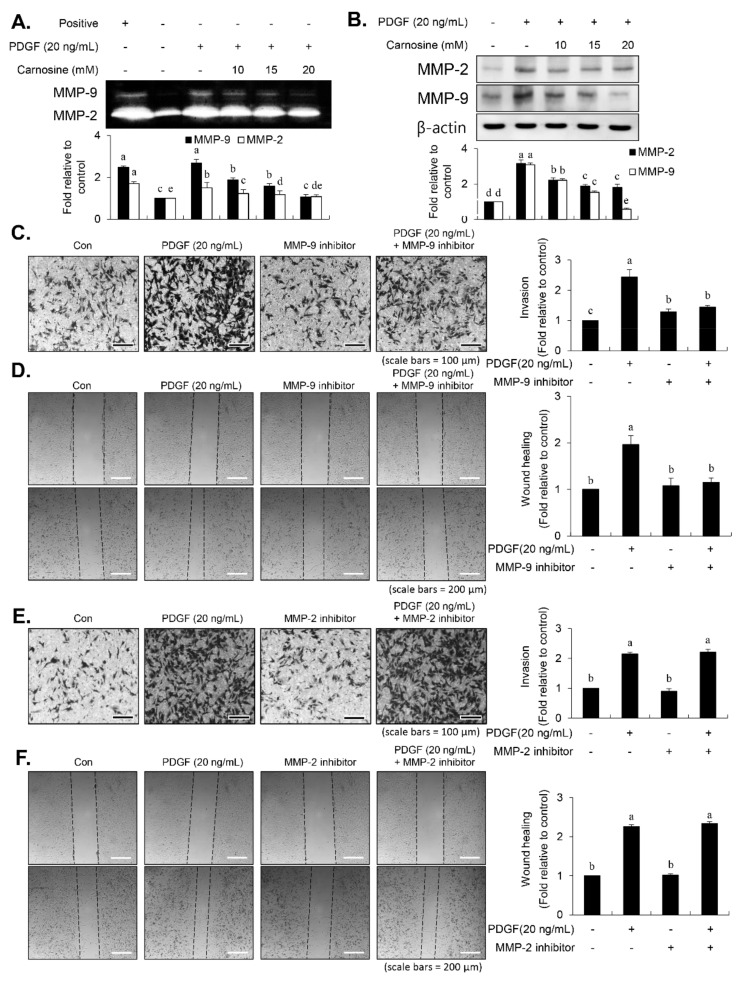
Carnosine suppressed PDGF-induced migration of VSMCs via reducing the matrix metalloproteinase (MMP)-9 activity. Serum-starved VSMCs were pre-incubated with carnosine (10, 15, and 20 mM) for 45 min, followed by reaction with PDGF (20 ng/mL) for 24 h. (**A**) Conditioned medium was evaluated for the gelatinase activities of MMP-2 and MMP-9 via gelatin zymography. The culture medium containing 10% FBS was used as positive control (Positive). (**B**) Cell lysates were employed for the immunoblot analysis. (**C**–**F**) Serum-starved VSMCs were pre-incubated with either MMP-2 inhibitor (10 nM) or MMP-9 inhibitor (10 nM) for 45 min, followed by PDGF treatment (20 ng/mL) for 24 h. Then, transwell plate invasion (**C**,**E**) and wound-healing assay (**D**,**F**) was performed. All data are expressed as the mean ± SE values of three experiments. The different letters indicate significant differences between the groups (*p* < 0.05).

**Figure 9:**
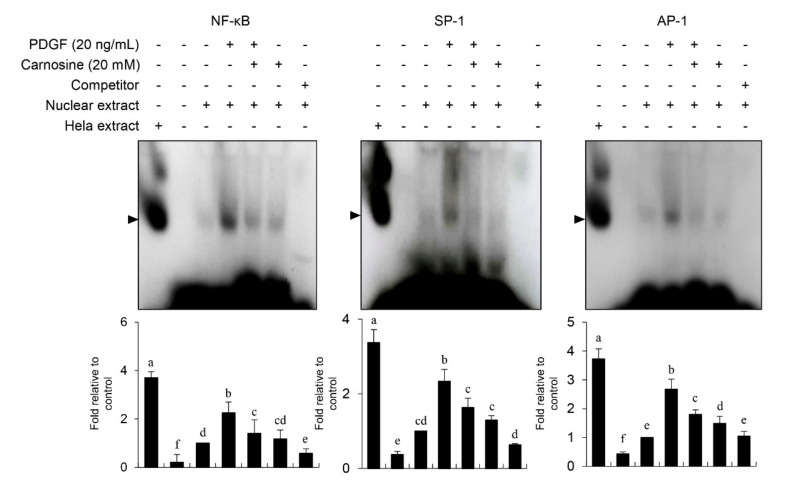
Carnosine disrupted MMP-9 activity by reducing the binding activities of Sp-1, AP-1, and NF-κB motifs in PDGF-stimulated VSMCs. The nuclear extracts were collected and assayed for the binding activities of Sp-1, AP-1, and NF-κB using EMSA with radiolabeled oligonucleotide probes. The quantified data from representative blots and plots are indicated. All data are expressed as the mean ± SE values of three experiments. The different letters indicate significant differences between the groups (*p* < 0.05).

VSMC migration is deeply involved in the expression of MMP-9, which is a proteolytic enzyme that degrades the extracellular matrix [9,11,12,13,14]. Next, we investigated the effect of carnosine on expression level of MMP-9 in PDGF-treated VSMCs using gelatin zymographic assay and immunoblot analysis. PDGF treatment revealed an upregulation in the levels of MMP-2 and MMP-9, as evidenced by gelatin-degrading activity and protein level (Figure 8A,B). However, the MMP-9 level was not enhanced by PDGF in the presence of carnosine (Figure 8A,B). Similar results were obtained with regard to MMP-2 (Figure 8A,B). We next investigated the role of MMP-9 activity in PDGF-induced migration of VSMCs using MMP-9 inhibitor. MMP-9 inhibitor suppressed the migration of VSMCs induced by PDGF as evidenced by transwell migration assay and wound-healing assay (Figure 8C,D). However, MMP-2 inhibitor treatment did not affect the PDGF-stimulated migration of VSMCs (Figure 8E,F), suggesting that MMP-9 is the key molecule to mediate PDGF-induced VSMC migration. These results demonstrated that carnosine might inhibit the PDGF-induced migration of VSMCs at least in part via blocking of MMP-9 expression. For investigating the regulatory mechanism of the carnosine-mediated reduction in MMP-9 activity in PDGF-treated VSMCs, binding abilities to AP-1, NF-κB, and Sp-1 motifs were examined using EMSA assay. PDGF treatment enhanced the AP-1, NF-κB, and Sp-1 binding activities in the nuclear extract from VSMCs (Figure 9). Pre-incubation of VSMCs with carnosine impeded the enhanced transcriptional binding of AP-1, NF-κB, and Sp-1 motifs in response to PDGF (Figure 9). These results suggested that the reduction in AP-1, NF-κB, and Sp-1 transcription factors is involved in carnosine-mediated repression of MMP-9 activity in PDGF-treated VSMCs.

### 3.8. Carnosine Attenuated PDGF-Stimulated Sprout Outgrowth of VSMCs Using an Ex Vivo Aortic Ring

**Figure 10:**
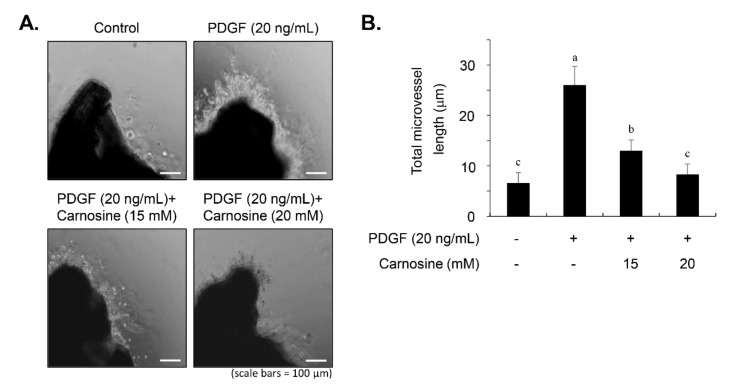
Carnosine attenuated the PDGF-stimulated sprout outgrowth formation of VSMCs from aortic rings. Sprout outgrowth of VSMCs was evaluated using aortic ring assay ex vivo. Aortic rings derived from rats were placed and embedded in Matrigel in 24-well plates. Aortic rings were incubated with the indicated concentrations of carnosine for 45 min and then stimulated with PDGF (20 ng/mL) for 5 days. (**A**) The sprout outgrowth responses were observed on light microscopic images. (**B**) Quantitative analysis of the aortic ring assay was performed by measuring sprout length. Bar graph shows the fold change in expression compared with the control. The values are expressed as the mean ± SD values of three independent experiments. The different letters indicate significant differences between the groups (*p* < 0.05).

To explore whether carnosine exerts anti-proliferative and anti-migratory effects on VSMCs to alleviate atherogenic activity, we performed an aortic ring assay ex vivo. As depicted in Figure 10A,B, PDGF treatment increased the length of sprout outgrowth. Carnosine addition led to a significant decline in PDGF-induced aortic sprouting of cells (Figure 10A,B). The results of an ex vivo aortic ring animal model revealed that carnosine significantly reduced induction of sprout outgrowth in PDGF-treated VSMCs.

## 4. Discussion

Carnosine, a dipeptide synthesized by our body, has been suggested to exhibit various physiological functions including anti-cancer, ant-diabetic, and anti-neurological effects [18]. However, the potential molecular mechanism of carnosine that underlies the suppressive effects of VSMC proliferation and migration remains unknown. In this study, we demonstrated that carnosine inhibited PDGF-induced proliferation of VSMC via p21WAF1-mediated arrest of G1-phase cell cycle and downregulation of JNK signaling. Additionally, carnosine suppressed PDGF-induced VSMC migration by downregulating transcription factor-associated MMP-9 expression. Furthermore, using an ex vivo animal model, we showed that carnosine impeded the PDGF-stimulated outgrowth vessel sprouting of VSMCs.

Abnormal proliferation of VSMC is a crucial step for the progression of vascular remodeling resulting in cardiovascular diseases such as re-stenosis and atherosclerosis [2,3,4,5]. PDGF is a potent stimulator that induces VSMC proliferation through the progression of cell cycle phases and phosphorylation of the signaling pathway [6,7,8,9]. Thus, developing natural agents that suppress PDGF-stimulated VSMC proliferation signaling might be a beneficial strategy for the treatment of proliferative vascular diseases. Results from our study indicated that carnosine inhibited PDGF-induced proliferation of VSMC, as evidenced by the MTT and cell counting assays. VSMCs maintain a quiescent status and a low proliferation level in the normal arterial media, thereby largely arresting the cells in the G1-phase cell cycle [8,9]. After vascular injury, VSMCs experience the phenotypic changes, which result in cell cycle transition [8,9]. Similarly, upon PDGF treatment, VSMCs proliferate and migrate from media to the intimal layer in the artery wall, where the cells stimulate cell cycle transition from the G1- to S-phase [8,9]. Here, we found that carnosine induced G1-phase arrest of the cell cycle in PDGF-induced VSMCs. These results demonstrate that carnosine repressed PDGF-stimulated VSMC proliferation via induction of G1-phase cell cycle arrest.

Cell cycle progression is tightly controlled by the positive cell cycle regulators, CDKs and cyclins [8,9]. The results from the present study showed that carnosine treatment suppressed the PDGF-induced expression of both CDKs (CDK2 and CDK4) and cyclins (cyclin E and cyclin D). Because the activities of CDKs and cyclins were negatively controlled by CDKIs [8,9], we investigated whether carnosine influences the expression level of p21WAF1 in PDGF-treated VSMCs. In fact, it has been previously reported that p21WAF1 functions in dual roles as evidenced by either suppression of cell growth [9,21] or promotion of cell proliferation [22,23,24,25]. As a positive influence, the results of the present study revealed an increased p21WAF1 expression in response to PDGF in VSMCs. PDGF-induced expression of p21WAF1 was further enhanced in the presence of carnosine, which has as a negative impact. Considering the expression of p21WAF1 as demonstrated in the current study, our data suggest that p21WAF1 is important for mediating a proliferative response in PDGF-treated VSMCs, whereas p21WAF1 also contributes to the carnosine-promoted suppressive role of cell proliferation in response to PDGF. However, both p27KIP1 and p53 levels were not influenced by the treatment with carnosine in PDGF-treated VSMCs. Thus, these results indicate that the suppressive effect of carnosine in PDGF-induced proliferation of VSMCs can be attributed to the p21WAF1-mediated G1-phase arrest of the cell cycle by inhibiting both CDK and cyclin expression.

Enhanced signaling, including MAPK and AKT pathways, impacts the proliferation of VSMCs, resulting in vascular remodeling and intimal thickening [6,7,8]. We found that PDGF-stimulated JNK signaling in VSMCs was inhibited by carnosine treatment. However, unexpectedly, the phosphorylation of p38MAPK, ERK1/2, and AKT by PDGF remained unaltered in the stimulation of carnosine. The role and importance of JNK signaling has been identified in PDGF-induced VSMC proliferation both in the in vitro and in vivo models [26,27]. Inhibition of JNK signaling has been suggested to be one of the main targets to consider while developing agents for the treatment of vascular diseases [26,27]. Furthermore, in line with our signaling data from immunoblot analysis, the transcriptional activity of AP-1, a JNK downstream effector, was blocked by treatment with carnosine in PDGF-treated VSMCs (Figure 7). Our results suggest that JNK signaling is at least in part correlated with the carnosine-mediated preventive effect in PDGF-stimulated VSMCs.

After vascular injury, besides the proliferation of VSMCs, VSMC migration showed phenotypic modulation that resulted in ECM degradation and vascular wall remodeling associated with vascular disorders [2,3]. MMP-9 expression is essential for ECM remodeling resulting from VSMC migration [8,10,11,12,13]. Carnosine treatment prevented the migration and invasion of VSMCs induced by PDGF. The result from the present study demonstrated that pretreatment with carnosine impeded the increased MMP-9 expression in PDGF-treated VSMCs. In addition, suppression of MMP-9, not MMP-2 inhibition, blocked the PDGF-induced migration of VSMCs. Moreover, the transactivation of transcription factors, namely AP-1, NF-κB, and Sp-1, existing in the promoter region of the MMP-9 gene was suppressed by carnosine in PDGF-induced VSMCs. Presumably, our data may suggest that carnosine inhibited PDGF-induced expression of MMP-9 by suppressing the binding activities of AP 1, NF κB, and Sp 1 motifs, leading to the repression of both ECM breakdown and VSMC migration. Finally, the PDGF-stimulated proliferation and migration of VSMCs were confirmed by evaluating the extent of vessel sprouting in the ex vivo aortic ring model. The length of vessel sprouting outgrowth in PDGF-stimulated VSMCs was interfered by pretreatment with carnosine. In accordance with the present results regarding the inhibition of proliferation and migration of VSMC, our ex vivo results imply that carnosine reduced the outgrowth region of vessel sprouting.

Many studies have been demonstrated that JNK signaling contributed to the regulation of cell function, including differentiation, apoptosis, migration, and proliferation [6,7,8,26,27,28]. Inhibition of JNK signaling by small molecule inhibitor has been attracted attention for therapeutic target to treat several diseases, such as cancer, obesity, and inflammation [28,29,30]. SP600125, the most commonly used chemical inhibitor of JNK signaling, has been shown evident anti-tumor potential in several cancers [28,29,30]. In our study, inhibition of JNK signaling by SP600125 suppressed the proliferation of VSMCs in response to PDGF. SP600125 treatment resulted in the reversion of PDGF-stimulated reduction of VSMC population at the G1-phase of the cell cycle. In addition, SP600125 treatment inhibited the migration of VSMC stimulated by PDGF. Our findings indicated that the inhibition of JNK phosphorylation could lead to the same phenotype as that observed with carnosine when measuring the cell number, cell viability, cell cycle arrest, and migration. The results of the present study suggest that JNK signaling might be involved in the preventive effect of carnosine in PDGF-stimulated VSMCs.

Taken together, our results demonstrated that carnosine impeded the PDGF-stimulated VSMC proliferation and migration. The carnosine-mediated suppression of PDGF-induced VSMC proliferation was caused by the p21WAF1-associated G1-phase arrest of the cell cycle via inhibition of both CDKs and cyclins expression. Moreover, carnosine disrupted PDGF-induced MMP-9 activity by decreasing the binding activity of Sp-1, NF-κB, and AP-1 motifs, which in turn downregulated VSMC migration. Additionally, JNK was identified as a critical signaling molecule partly involved in carnosine-mediated anti-proliferative and anti-migratory mechanisms in PDGF-stimulated VSMCs. Furthermore, the attenuation of PDGF-induced blood vessel sprout outgrowth in VSMCs was verified using the aortic ring assay ex vivo. Therefore, these results might suggest the promising potential of carnosine for its preventive and therapeutic effects against cardiovascular diseases.
